# Structure and properties of composite surface layers produced on NiTi shape memory alloy by a hybrid method

**DOI:** 10.1007/s10856-018-6118-5

**Published:** 2018-07-17

**Authors:** Justyna Witkowska, Agnieszka Sowińska, Elżbieta Czarnowska, Tomasz Płociński, Bogusław Rajchel, Michał Tarnowski, Tadeusz Wierzchoń

**Affiliations:** 1 Faculty of Materials Science and Engineering, Warsaw University of Technology, Wołoska 141, 02-507 Warsaw, Poland; 2 Pathology Department, Children’s Memorial Health Institute, Dzieci Polskich 20, 04-730 Warsaw, Poland; 3 Institute of Nuclear Physics Polish Academy of Sciences, Radzikowskiego 152, 31-342 Cracow, Poland

## Abstract

A hybrid process that combines oxidation under glow-discharge conditions with ion beam-assisted deposition (IBAD) has been applied to mechanically polished NiTi shape memory alloy in order to produce composite surface layers consisting of a TiO_2_ layer and an external carbon coating with an addition of silver. The produced surface layers a-C(Ag) + TiO_2_ type have shown increased surface roughness, improved corrosion resistance, altered wettability, and surface free energy, as well as reduced platelet adhesion, aggregation, and activation in comparison to NiTi alloy in initial state. Such characteristics can be of great benefit for cardiac applications.

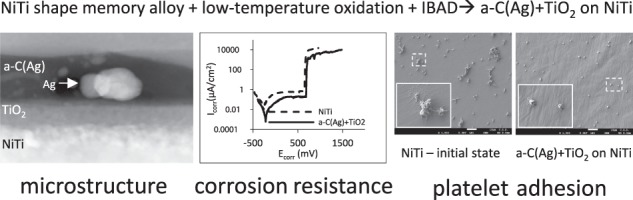

## Introduction

Constantly rising demand for cardiovascular implants makes it necessary to develop new material solutions to meet surgical procedures, shorten the time of tissue healing and patient recovery, as well as to minimize post-surgical complications. For this reason, so-called “smart materials” are being used more and more often in designing new generation implants. It is assumed that their global share will increase by 10.2% annually, reaching a value of $42.2 billion in 2019 [1]. One such material, NiTi alloy, which is gaining in popularity, is characterized by a set of unique properties, which include shape memory effect and superelasticity. It is used, among others, in bone implants such as clamps for spinal correction, plates for osteosynthesis, orthodontic wires or cardiovascular implants, including self-expanding stents [2].

The main limitation in using NiTi shape memory alloys for medical applications is their high nickel content. Released from the surface of the material into the human body, it can have a toxic effect on the surrounding tissues [3, 4], induce allergic reactions and even lead to cancer in the long term [5]. NiTi shape memory alloy is a self passivating material with relatively good corrosion resistance due to the thin layers of titanium oxide spontaneously produced on the alloy’s surface [6, 7]. However, they are insufficient in providing adequate protection when used in medical implants [8, 9] because of their fragility and poor self-healing properties [10, 11]. A damaged passive layer can increase the release of nickel ions into the biological environment [11]. Therefore, increasing the biocompatibility of NiTi is necessary, which can be achieved through various surface engineering methods [12–19]. But not all of the methods used allow for the retention of NiTi’s specific properties, as they can become impaired by the use of high temperatures. It was noted that above 300 °C, precipitations of the Ni_4_Ti_3_ phase are observed in the alloy structure, that affects shape memory and superelasticity [20].

Our previous studies showed that glow-discharge treatments in low-temperature plasma such as nitriding [21], oxidation [22] or oxynitriding [23], allow for the formation of surface layers that increase the corrosion resistance and biocompatibility of NiTi. This is due to the high chemical affinity of titanium to oxygen or atomic nitrogen. What is more, the RF CVD process enables the formation of an amorphous carbon coating on the surface, which gives the material athrombogenic properties—a feature necessary when used in contact with blood [22, 23].

Another problem associated with the use of implants are bacterial infections following surgery [24, 25]. Prophylactic treatment by antibiotics is recommended but becoming less effective because of the resistance various strains of bacteria develop with regard to antibiotics [26]. To meet these challenges and provide antimicrobial action, it is proposed to add silver or silver nanoparticles to surface coatings [27, 28]. For this reason we decided to enhance the previously described methods of hybrid surface treatment and combine low-temperature oxidation under glow-discharge conditions with ion beam-assisted deposition, which produces a silver nanoparticle-enriched amorphous carbon coating on top of the titanium oxide layer. In the first stage of research, we decided to examine this surface layer in the same scope as in our previous studies [22, 23] to know if it met the basic properties required of materials that come in contact with blood.

Hence, the aim of this study was to produce an a-C(Ag) + TiO_2_ type composite surface layer and verify its structure and properties in terms of its application in cardiological implants.

## Materials and methods

### Materials

The investigated material was NiTi shape memory alloy (50.8% at Ni) in the form of Φ8 × 1 mm thick discs obtained by cutting from a rod. Before deposition, the specimens were ground to a 2400 grit finish and washed in an ultrasonic bath. The glow-discharge oxidation process was conducted at a temperature of 300 °C, controlled by a thermocouple and at a working chamber pressure of 1.6 mbar in an atmosphere of pure oxygen for 30 min. A silver nanoparticle-enriched amorphous carbon coating was produced on the oxidized layer via dual beam ion beam-assisted deposition (IBAD). The graphite plate (50 × 50 mm) with a centrally positioned vertical strip of Ag (2 × 50 mm) was bombarded by a 15 keV Ar^+^ ion beam at an angle of 20° in relation to the surface. Both the ions and atoms of carbon and silver were knocked out and directed towards the surface of the coated sample placed in the head of the 3-axial goniometer. At the same time, the dynamically increasing a-C(Ag) coating was additionally bombarded with a 15 keV Ag^+^ ion beam. In order to ensure optimum homogeneity of the coating, the specimen was repeatedly shifted across the additional beam of Ag^+^ ions. The process was conducted in a vacuum of about 10^−6^ mbar.

### Structure of the layer

The cross-section microstructure of the surface layers was observed by a high-resolution scanning transmission electron microscope (HRSTEM) working under an acceleration voltage of 200 keV (HD2700, Hitachi, Tokyo, Japan) with bright field and high-angle annular dark field detectors. Specimens measuring 15 × 3 × 5 μm were prepared for microscopic observations by means of the focused ion beam lift-out technique and an ion scanning microscope and thinned to approximately 100 nm by a gallium ion beam with an energy of 40 kV. Additionally, the linear distribution of elements along the produced surface layer was determined.

### Surface characteristics

#### Concentration of bonds

To determine the relative concentration of bonding with sp3/sp2 hybridizations, confocal Raman dispersive microspectroscopy was used. A beam of light at a 532 nm wavelength and a power of 2.5 mW was directed towards a thin zone of the surface carbon coating and Raman scattered photons were recorded. The recorded Raman spectra were then analyzed using the OMNIC module from the PeakFit program (Thermo). The graph (Fig. 2) shows both the recorded experimental spectrum and the fractional spectra obtained by numerical analysis.

#### Topography

Surface topography examinations were conducted with the use of a Wyko NT 9300 scanning optical profilometer and a Vecco atomic force microscope with a Multimode VIII controller (tapping mode, tip model ACSTA, AppNano, CA, USA). Roughness parameters were measured from 480 × 640 μm areas (optical profilometer) and 10 × 10 μm (atomic force microscope) areas. Three different measurements were done for each sample and the results were presented as mean ± standard deviations.

#### Contact angles and surface energy

The wettability was assessed using a goniometer—Contact Angle System OCA 20 (DataPhysics, CA, USA) at room temperature. The measurements were conducted with the use of distilled water and diiodomethane. A drop of 0.4 μl was applied to the surface of each sample and its picture was captured immediately afterwards. The images of the droplets were analyzed by SCA20 software. Ten measurements were made for each sample and the average values and standard deviations were calculated. The surface-free energy was calculated for each sample using the Owens–Wendt standard method [29] based on the obtained values of contact angles for both used liquids.

### Corrosion resistance

The corrosion resistance was assessed by potentiodynamic measurements with the use of an Autolab PGSTAT 100 potentiostat. The measurements were performed for the NiTi alloy in initial state and after surface treatment at a temperature of 37 °C in Ringer’s solution consisting of 7.0 g/dm^3^ NaCl, 0.075 g/dm^3^ KCl, 0.1 g/dm^3^ CaCl2·2H_2_O and 0.1 g/dm^3^ NaHCO_3_ with a chloride ion content of 4.33 g/dm^3^. The specimens were immersed in a corrosive solution in current-free conditions for approximately 2 h. Potentiodynamic examinations were conducted in a three-electrode setup with the test material being the working electrode, a saturated calomel electrode as the reference and a platinum electrode as the auxiliary electrode at a potential range from approximately 150 mV below the stabilized open circuit potential up to 1500 mV. The tested material was polarized at a constant potential sweep rate of 0.2 mV/s. Corrosion current density and the corrosion potential were determined using the Tafel extrapolation method.

### Tests with PRP

Assessment of blood platelet adhesion, aggregation, and activation was performed with the use of human platelet-rich plasma (PRP), obtained from the blood of healthy donors, according to method described in ref. [22]. The prepared specimens were tested under a scanning electron microscope (JSM-7600F, JEOL) under an acceleration voltage of 5 kV in LEI mode (lower secondary electron image). The number of platelets and their aggregates were measured using morphometric CellSens (Olympus, Germany) software.

### Statistical analyses

Results were presented as mean ± standard deviation. Statistical analyses were performed using the Fisher and Student’s *t*-tests at a significance level of *α* = 0.05.

## Results

### Structure of the layer

**Fig. 1:**
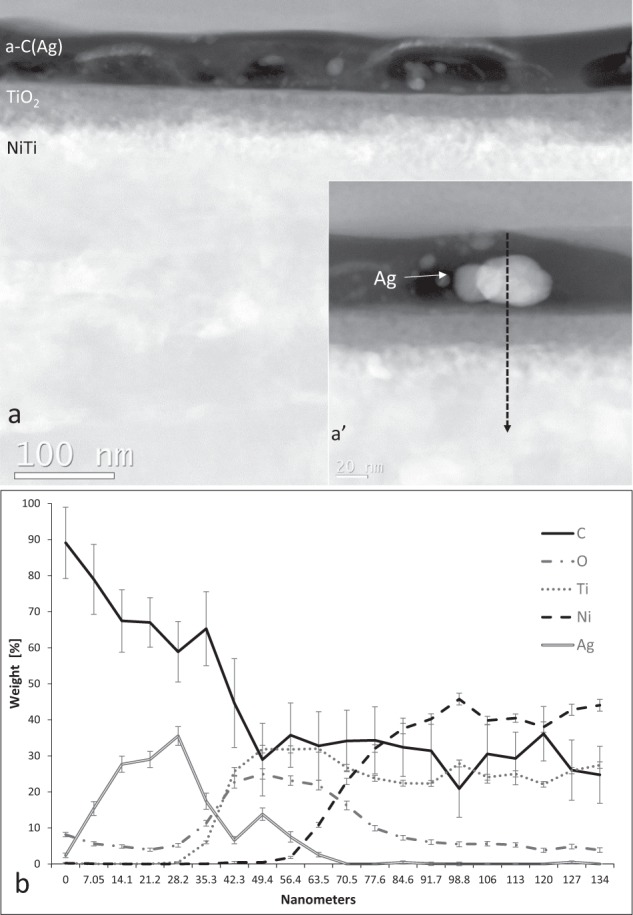
Microstructure of an a-C(Ag) + TiO_2_ type surface layer produced on NiTi in a hybrid process: **a** cross-section of the layer by ZC-STEM—atomic mass contrast; **a**' magnification of silver nanoparticles present in the carbon coating; **b** linear distribution of elements across the cross-section of the surface layer with error bars marked

From transmission electron microscope observations comes that the hybrid layer produced on NiTi shape memory alloy is an a-C(Ag) + TiO_2_ type (Fig. 1a). An outer zone consists of amorphous carbon coating with a thickness of about 40 nm, enriched with randomly distributed silver nanoparticles with a diameter of about 10 nm (Fig. 1a'). Below, a titanium oxide layer with a thickness of about 30 nm was present. As shown in our previous studies, the TiO_2_ produced under glow-discharge conditions is rutile with a nanocrystalline structure [22, 30, 31]. The distribution of elements along the cross-section did not show the presence of nickel in the subsurface layer (Fig. 1c).

### Surface characteristics

#### Concentration of bonds

**Fig. 2:**
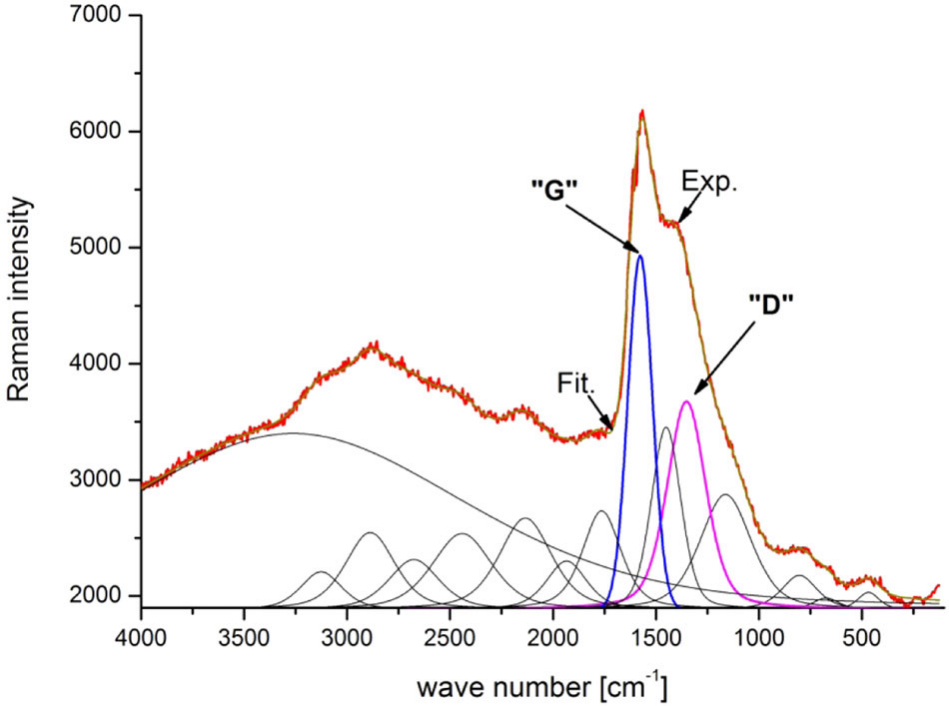
Raman spectrum of a-C(Ag) coating with a recorded experimental (Exp.) spectrum and fractional spectra obtained by numerical analysis

Investigation of the bonding structure with Raman spectroscopy showed the typical D and G bands of amorphous carbon (Fig. 2). A numerical analysis of the results indicates that the ratio of sp3 to sp2 bonds is about 40%.

#### Topography

**Fig. 3:**
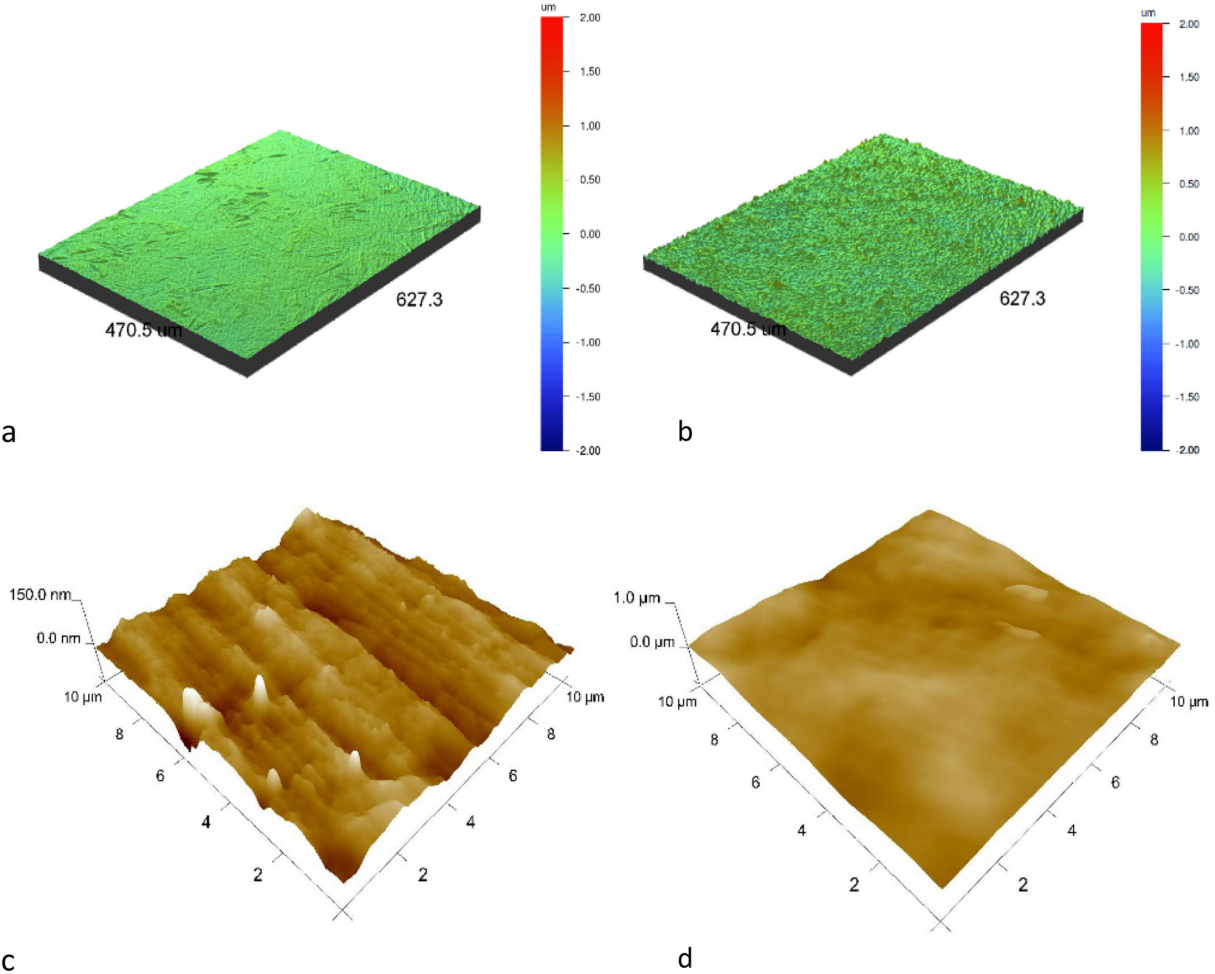
Surface topography observed by: **a**, **b** an optical profilometer and **c**, **d** AFM. NiTi in initial state (**a**, **c**) and with an a-C(Ag) + TiO_2_ surface layer (**b**, **d**)

Surface topography observations showed an increase in surface roughness following oxidation and carbon coating ion beam-assisted deposition both in the micro-scale (optical profilometer) and in the nanoscale (AFM) (Table 1 and Fig. 3). Surface development can be the result of both titanium dioxides produced in the oxidation process and the nature of the IBAD process, especially the additional bombardment with the Ag^+^ ion beam during production of the carbon coating. This trend stands in contrast to our previous experience with coatings produced using the RF CVD method that rather smoothed or slightly developed the treated surfaces [22, 23].

**Table 1:** Surface roughness (*n* = 3) of NiTi alloys in their initial state and after the hybrid process (a-C(Ag) + TiO_2_ surface layer)

Methods	Sample	*R*_a_	*R*_q_	*R*_z_
Optical profilometer [µm]	NiTi	0.040 ± 0.001	0.052 ± 0.002	0.499 ± 0.031
	a-C(Ag) + TiO_2_	0.108 ± 0.003	0.142 ± 0.008	1.753 ± 0.025
AFM [nm]	NiTi	0.012 ± 0.001	0.016 ± 0.001	0.176 ± 0.044
	a-C(Ag) + TiO_2_	0.044 ± 0.016	0.055 ± 0.017	0.344 ± 0.016

*R*_a_ roughness average, *R*_q_ root mean square roughness, *R*_z_ mean height of the five highest peaks above the average line decreased by an average of the five lowest valleys below the average

#### Contact angles and surface energy

The contact angles for both liquids i.e., distilled water and diiodomethane increased after surface treatment. However, a statistically significant difference was noted only for distilled water. The surface free energy values calculated using the Owens–Wendt method [29] were lower for the a-C(Ag) + TiO_2_ layer. The ratio of the dispersive component to the polar component increased for the samples with a surface layer. All the results are presented in Tables 2 and 3.

**Table 2:** Contact angles measured for NiTi in initial state and with an a-C(Ag) + TiO_2_ surface layer with the use of distilled water and diiodomethane (*n* = 10)

Contact angle [°]		
	NiTi	a-C(Ag)+TiO_2_
Distilled water	80.5 ± 4.2	95.8 ± 0.7
Diiodomethane	48.8 ± 0.9	50.6 ± 2.5

**Table 3:** Surface-free energy values calculated for NiTi in initial state and with an a-C(Ag) + TiO_2_ surface layer

Surface-free energy		
	NiTi	a-C(Ag)+TiO_2_
γ [mN/m]	36.04	32.84
γ_d_ [mN/m]	32.14	32.56
γ_p_ [mN/m]	3.91	0.28

*γ* total surface free energy, *γ*_d_ dispersive component of surface free energy, *γ*_p_ polar component of surface free energy

### Corrosion resistance

**Fig. 4:**
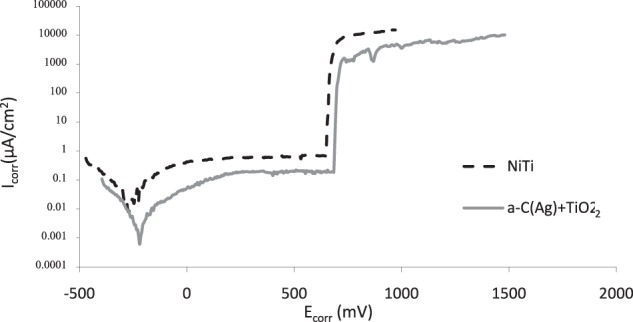
Electrochemical measurements: potentiodynamic curves of NiTi alloy in its initial state and with an a-C(Ag) + TiO_2_ surface layer after exposure to Ringer’s solution

Potentiodynamic measurements showed an improvement of corrosion resistance of the samples with a composite a-C(Ag) + TiO_2_ surface layer in comparison with the NiTi alloy in its initial state. The shape of the potentiodynamic curves indicated the increased stability of NiTi in a corrosive environment following surface treatment processes, as indicated by a smaller current in the passive zone for the treated samples (Fig. 4). The obtained electrochemical parameters (Table 4) also confirmed an increase in the corrosion potential and break-down potential values and a decrease of one order of magnitude in the corrosion current values for the samples with the surface layer.

**Table 4:** Characteristic electrochemical values of NiTi alloy in its initial state and with an a-C(Ag) + TiO_2_ surface layer after exposure to Ringer’s solution

Sample	*E*_corr_ [mV]	*I*_corr_ [µA/cm^2^]	*E*_bd_ [mV]	*I*_bd_ [µA/cm^2^]
NiTi	–266	1.39E-02	648	6.77E-01
a-C(Ag) + TiO_2_	–220	5.01E-03	684	1.83E-01

*I*_corr_ corrosion current density, *E*_corr_ corrosive potential; *E*_bd_ break-down potential, *I*_bd_ break-down current density

### Tests with PRP

**Fig. 5:**
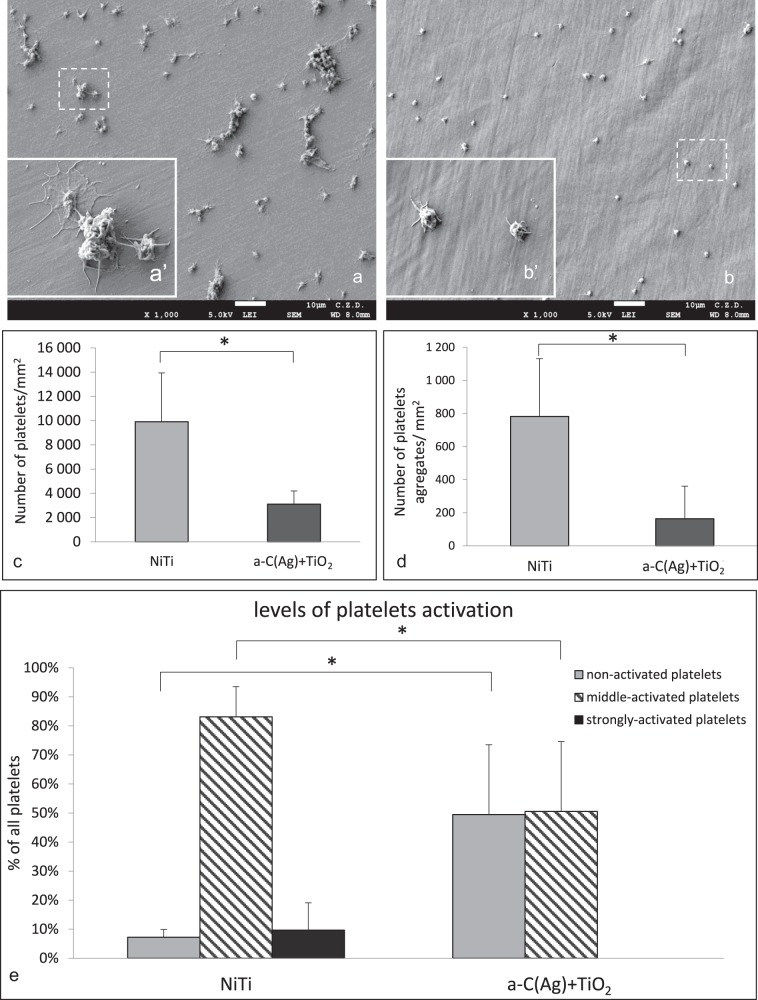
Platelet adhesion, aggregation, and activation on the surfaces of the test materials. Platelets on: **a**, **a**' NiTi in initial state and **b**, **b**' with an a-C(Ag) + TiO_2_ surface layer. Quantitative assessment: **c** platelet adhesion (*n* = 5) and **d** platelet aggregates (*n* = 5). Platelet activation according to morphological features: **e** Quantitative assessment of a variety of platelet activity (*n* = 3); *p ≤ 0.05 NiTi vs. a-C(Ag) + TiO_2_

Platelet incubation on the surface of the samples revealed statistically significant differences in the number of adhering platelets and their aggregates for NiTi alloy in initial state (Fig. 5a) compared with an a-C(Ag) + TiO_2_ layer (Fig. 5b). Lower adhesion and aggregation was noted for the material after the hybrid process (Fig. 5c, d). It is important to note that the platelets in the population on the carbon coating surface included both non-activated and middle-activated cells, while platelets with more heterogeneous activity were present on the NiTi alloy without a surface layer and included strongly activated cells (according to the description by Goodman et al. [32]) (Fig. 5e). According to Cooper’s classification scheme [33] the platelets on the NiTi with surface layer were in I and II stages of activation and the platelets on the NiTi without surface layer were mostly in III stage, but cells in II, IV, and V stages were also observed in the population. What is more, in comparison to our previous studies when platelet adhesion was tested in the same conditions after incubation on a carbon coating produced on oxynitrided NiTi by the RF CVD method [23], the present results for coating produced in the IBAD process are more promising due to a lower amount of adhering platelets and their aggregates (an average of 8618 vs. 3109 platelets per mm^2^ and an average of 545 vs. 164 platelets aggregates per mm^2^).

## Discussion

In the present study, surface modification by means of the hybrid process consisting of low-temperature glow-discharge oxidation and ion beam-assisted deposition is proposed as a method to enhance the biocompatibility of NiTi shape memory alloy in terms of its use in cardiological implants. The described treatment led to produce a surface layer composed of a silver nanoparticle-enriched amorphous carbon coating and a nanocrystalline titanium oxide layer (a-C(Ag) + TiO_2_ as observed from the surface inwards). It is known that use of low-temperature process does not affect the specific properties of NiTi alloy. While produced surface layers improved the corrosion resistance of NiTi, which is key in limiting metallosis, i.e., the potential harmful effects of nickel being released from the surface. The produced surface layer altered the NiTi surface in the range of topography and wettability, made it more hydrophobic, which may have contributed to the limitation of platelet adhesion and aggregation. What is more, in comparison to our previous studies when platelet adhesion was tested in the same conditions after incubation on a carbon coating produced on oxynitrided NiTi by the RF CVD method [23], the present results for coating produced in the IBAD process are more promising due to a lower amount of adhering platelets and their aggregates (an average of 8618 vs. 3109 platelets per mm^2^ and an average of 545 vs. 164 platelets aggregates per mm^2^).

Numerous surface engineering methods are successfully used to improve the resistance of NiTi alloys in corrosive environments [12–19]. However, to the best of our knowledge, this is the first time the combination of low-temperature oxidation and IBAD has even been proposed. The presented corrosion resistance test results are in accordance with our previous studies, which revealed improved corrosion resistance for layers oxidized under glow-discharge conditions, further enhanced after adding a carbon coating to the surface [34]. However, the electrochemical parameters are not as high as for the carbon coating produced by the RF CVD method. Referring to Sui et al. [35] this may be due to a different conductivity of the carbon layers, which may have an influence on the electron transportability on the surface of the sample that occurs in electrochemical corrosion. However, we have not investigated the conductivity of any of the coatings, so we are unable to confirm such dependencies.

Materials designed for contact with blood, in addition to providing high corrosion resistance, are required to reduce blood coagulation and thrombosis. Numerous studies indicate the multitude of factors that influence the interactions between the material and the body cells, among which the most important are chemical composition, wettability and surface energy, surface topography, charge, polarity etc. and the relationship between them [31, 36]. Carbon-based coatings are singled out as being capable of providing good hemocompatibility and reduce blood clotting in particular. The most commonly produced coatings include: a-CH type amorphous carbon coatings [37–39], nanocrystalline diamond coatings [40] and carbon nanotubes [39]. Presumably, chemical composition plays a major role in reducing platelet adhesion. However, its correlation with other factors has to be taken into account [40].

Among other surface topography, especially in nanoscale is important feature that influences cell-material interaction [37, 39, 41]. Its effect on platelet adhesion and aggregation is a complex issue, and literature reports still do not indicate, which topography is most optimal for obtaining a non-thrombogenic surface. Karagkiozaki et al. [39] noted an increase in the hemocompatibility properties for rougher a-CH coating surfaces and for coatings with carbon nanotubes [41]. In contrast, Lopez-Santos et al. [37] observed enhanced platelet adhesion on surfaces with increased roughness. They associated it with albumin adsorption which, in their opinion, is facilitated on smoother surfaces and which in turn inhibits platelet adhesion. At the same time, there are studies that show a lack of influence of surface topography on the adhesion of blood platelets on the biomaterial surface [8, 40]. In the present study, the a-C(Ag) + TiO_2_ surface layer was shown to increase surface roughness, which turned out to have a favorable effect on NiTi in terms of its hemocompatibility. This is in contrast to our previous studies on a-CNH carbon coatings, where we observed reduced platelet adhesion or activation in the case of smoother surfaces [22] and surfaces that were more developed than in their initial state, but not as extensive as in this study [23].

The next influential factor is surface wettability and surface free energy [42]. In the present study, a more hydrophobic a-C(Ag) + TiO_2_ surface layer with lower surface free energy resulted in reduced platelet adhesion and aggregation, which is in accordance both with our previous studies [23] and selected literature reports [11, 43]. Researchers observed that water molecules are strongly bound to hydrophilic surfaces and are not easily displaced by absorbing proteins, whereas hydrophobic surfaces facilitate displacement. Further research including protein biofilm studies is needed to verify these observations.

New with regard to the previously described surface layers is the presence of silver nanoparticles in the outer zone of the composite surface layer. After adding silver to the amorphous carbon coating produced by the IBAD method, its athrombogenic properties were retained, and what is more, the number of platelets and their aggregates was even lower than in the previously presented coatings produced by RF CVD. Silver is known for its antibacterial properties and has been used for a variety of applications ranging from wound healing [44] to waste water treatment [45]. Although the effect on bacterial growth was not measured in this study, the literature allows us to assume that the produced a-C(Ag) + TiO_2_ type composite layer will provide an antimicrobial effect [27, 28] thus enhancing the biocompatibility of NiTi in medical applications. It is necessary to extend the study and investigate the effect of silver concentration in the coating on antibacterial properties and on cell growth (endothelial cells in particular). An optimal silver concentration should protect against bacterial growth without affecting human body cells.

This study has several limitations, among other lack of experiments which could: check if nickel ions are released from the modified surface, investigate the composition and distribution of proteins in biofilm on the surface of specimens to clear its effect on platelets decreased adhesion and activation, examine the influence of the a-C(Ag) + TiO_2_ layer on endothelial cell proliferation, and verify the antimicrobial effect of silver-containing coatings in the context of cardiovascular applications. Therefore it should be extended by further research.

## Conclusion

The presented surface treatment of NiTi shape memory alloy by low-temperature plasma oxidation under glow-discharge conditions combined with ion beam-assisted deposition yields a composite a-C(Ag) + TiO_2_ type surface layer (as observed from the surface inwards). NiTi shape memory alloy with a produced surface layer offers improved properties in comparison to the material in initial state in the scope of corrosion resistance and compatibility with blood. It can be concluded that obtained results are promising in terms of prospective use in cardiological implants and further research in this area is necessary.
